# Socioeconomic and Health Disparities between Transgender and Cisgender Older Adults in the Swedish Population

**DOI:** 10.1093/geroni/igaf122.101

**Published:** 2025-12-31

**Authors:** J Lucas Tilley, Ylva Moberg, Anyah Prasad, Emma Essen

**Affiliations:** Stockholm University, Stockholm, Stockholms Lan, Sweden; Stockholm University, Stockholm, Stockholms Lan, Sweden; Vanderbilt University, Boston, Massachusetts, United States; Uppsala University, Uppsala, Uppsala Lan, Sweden

## Abstract

Transgender older adults are an understudied group, and most current studies are based on convenient samples which can be subject to selection bias. Recruiting a sizeable representative sample of transgender older adults can be difficult and expensive as they are a very small percentage of the population and hard to reach due to stigma, privacy, and trust issues. Population data from centralized administrative and medical health records available in some countries with universal health care are an under-tapped potential to understand aging among transgender individuals. In this study, we use 2019 data from the Swedish National Patient Register to identify individuals with a diagnosis of gender incongruence and get information on their past year health (hospitalization and anti-depression/anxiety medication usage). Their corresponding sociodemographic data were obtained from Statistics Sweden. We identified 535 transwomen and 206 transmen age 50 and above and each trans person was matched, based on their birth year, with 10 cisgender men and women each. Compared to cisgender men and women, transgender individuals were more likely to live in urban areas, less likely to be married, and tend to have lower earnings and retire early. In logistic regression, after controlling for sociodemographic factors, compared to cisgender men, transgender women and men experienced significantly higher odds of hospitalization (OR = 1.85., p < 0.001; OR = 1.88, p = 0.001, respectively) and were significantly more likely to be on anti-depression/anxiety medication (OR = 2.23, p < 0.001; OR = 3.04., p < 0.001, respectively). These findings highlight the need for targeted interventions to elevate the disproportionate burden of poor health among transgender older adults.

